# Supplementary material for: Annotation and Molecular Characterisation of the TaIRO3 and TaHRZ Iron Homeostasis Genes in Bread Wheat (Triticum aestivum L.)
Source: Genes (Basel). 2021 Apr 27;12(5):653. doi: 10.3390/genes12050653 (PMC8146704; doi:10.3390/genes12050653)
Supplement: Supplementary file 1 [file genes-12-00653-s001.zip › genes-1125300-supplementary.pdf]

genes
MDPI

Supplementary Materials

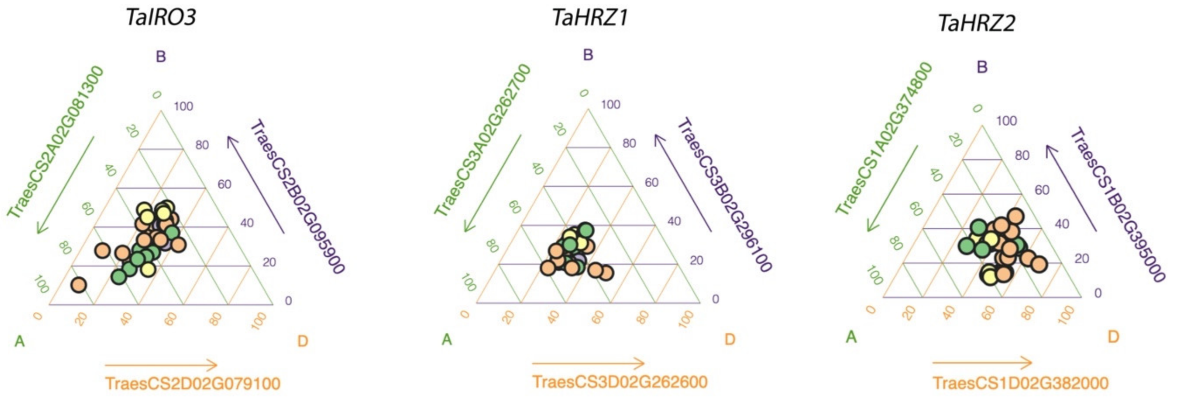

Supplementary Figure 1 Ternary plots showing expression levels of *TaIRO3*, *TaHRZ1*, and *TaHRZ2* genes from subgenomes A (green), B (purple), and D (orange) as extracted from the Wheat Expression Browser database (wheat-expression.com). TraesIDs of each *TaIRO3*, *TaHRZ1*, and *TaHRZ2* homoelog is provided alongside each ternary plot.

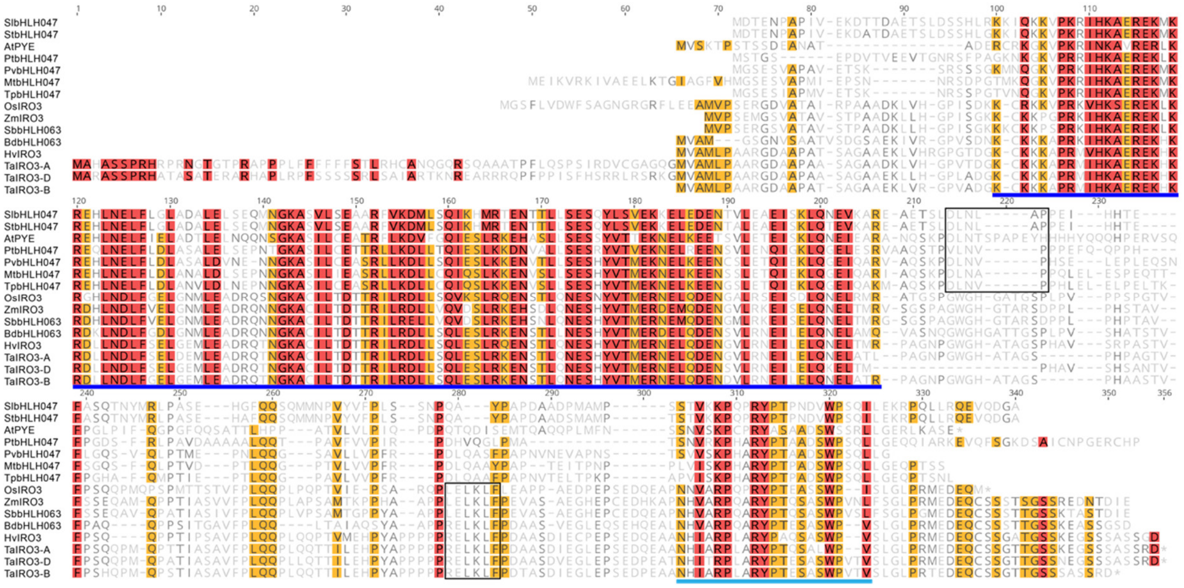

Supplementary Figure 2 Conserved amino acid sequences of IRO3/PYE proteins. Red shading represents 100% similarity. Orange shading represents 80-100% shading. Black numbers represent 60-80% similarity. Grey numbers represent less than 60% similarity. The Helix-Loop-Helix domain (dark blue) and a conserved region of unknown function (light blue) are underlined. Boxes highlight locations containing EAR motifs. *Arabidopsis thaliana* (At), *Brachypodium distachyon* (Bd), *Hordeum vulgare* L. (Hv), *Medicago truncatula* (Mt), *Oryza sativa* L. (Os), *Phaseolus vulgaris* L. (Pv), *Populus trichocarpa* (Pt), *Solanum lycopersicum* L. (Sl), *Solanum tuberosum* L. (St), *Sorghum bicolor* L. (Sb), *Trifolium pratense* L. (Tp), *Triticum aestivum* L. (Ta) and *Zea mays* L. (Zm).

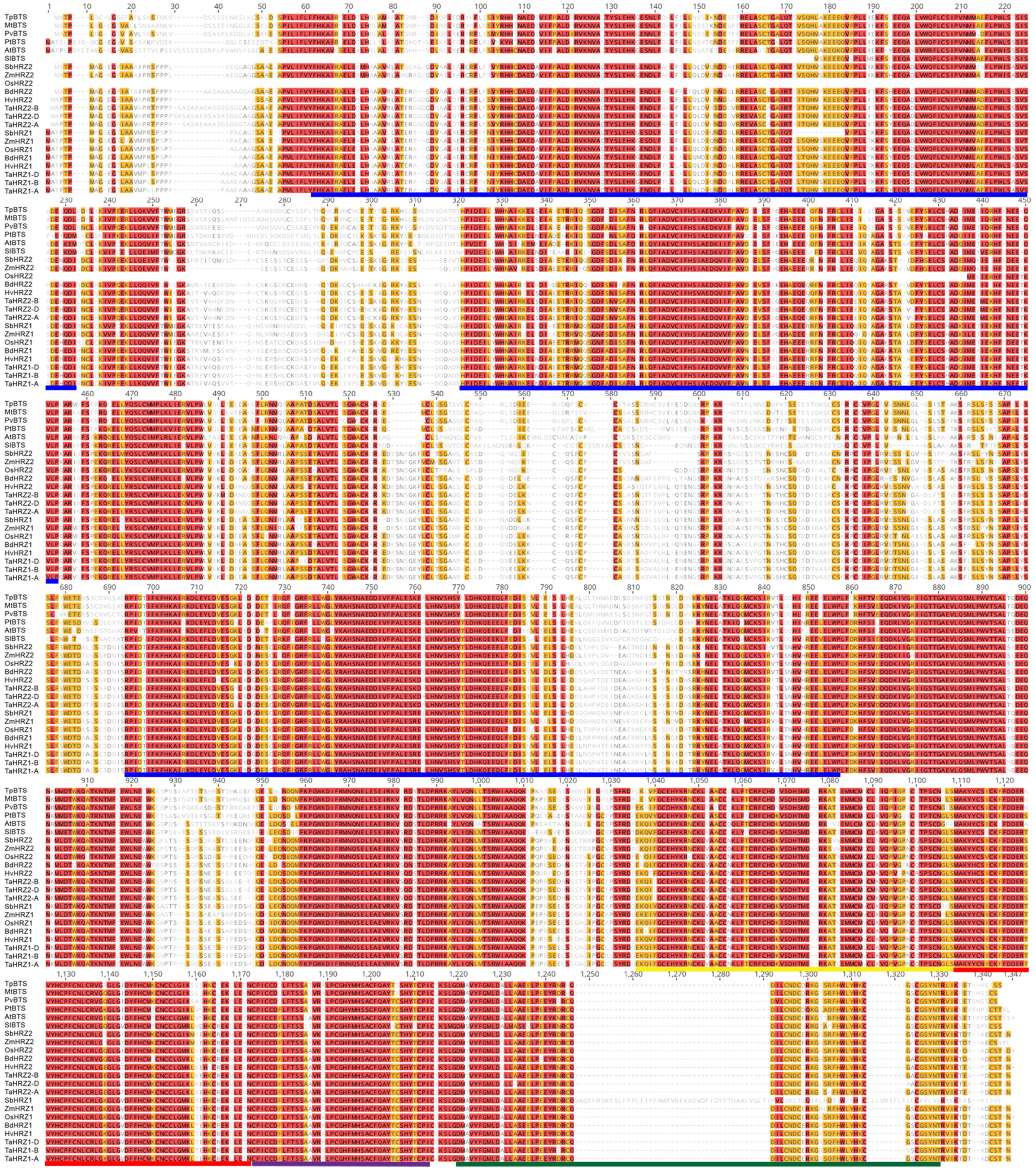

Supplementary Figure 3 Conserved amino acid sequences of HRZ1 and HRZ2 proteins. Red shading represents 100% similarity. Orange shading represents 80-100% shading. Black numbers represent 60-80% similarity. Grey numbers represent less than 60% similarity. The hemerythrin (blue), Znf CHY (yellow), Znf CTCHY (red), Znf RING (purple) and RCHY1 zinc-ribbon (green) domains are underlined *Arabidopsis thaliana* (At), *Brachypodium distachyon* (Bd), *Hordeum vulgare* L. (Hv), *Medicago truncatula* (Mt), *Oryza sativa* L. (Os), *Phaseolus vulgaris* L. (Pv), *Populus trichocarpa* (Pt), *Solanum lycopersicum* L. (Sl), *Sorghum bicolor* L. (Sb), *Trifolium pratense* L. (Tp), *Triticum aestivum* L. (Ta) and *Zea mays* L. (Zm).

Supplementary Table 1 Characteristics of *TaIRO3* and *TaHRZ* genes and *in silico* characterisation of TaIRO3 and TaHRZ proteins.

Gene	No. of splice variants	ID (Ensembl plants)	Molecular weight (Da)	No. of AA	Gravy score	Theoretical pI	Predicted Subcellular Localisation
TaIRO3-A	2	TraesCS2A02G081300.1	36657.12	338	-0.601	6.13	0.933 (Nucleus)
TaIRO3-B	1	TraesCS2B02G095900.1	29334.81	270	-0.642	5.33	0.9101 (Nucleus)
TaIRO3-D	1	TraesCS2D02G079100.1	36238.83	329	-0.699	8.99	0.8146 (Nucleus)
TaHRZ1-A	11	TraesCS3A02G262700.5	139307.38	1237	-0.335	5.52	0.4959 (Nucleus)<br>0.4316 (Cytoplasm)
TaHRZ1-B	15	TraesCS3B02G296100.7	139387.4	1237	-0.349	5.57	0.5106 (Nucleus)<br>0.4165 (Cytoplasm)
TaHRZ1-D	10	TraesCS3D02G262600.1	139367.49	1237	-0.335	5.58	0.4861 (Cytoplasm)<br>0.4131 (Nucleus)
TaHRZ2-A	3	TraesCS1A02G374800.2	139639.19	1239	-0.361	5.68	0.4743 (Nucleus)<br>0.3974 (Cytoplasm)
TaHRZ2-B	1	TraesCS1B02G395000.1	139842.38	1241	-0.372	5.66	0.5141 (Cytoplasm)<br>0.3408 (Nucleus)
TaHRZ2-D	2	TraesCS1D02G382000.1	139925.45	1242	-0.356	5.73	0.5111 (Cytoplasm)<br>0.3857 (Nucleus)

Supplementary Table 2 The orthologous IRO3/bHLH063/bHLH047/PYE and HRZ/BTS proteins included in phylogenetic analyses.

Orthologous protein	Gene ID or Location ID	Source	Length (aa)
OsIRO3	LOC_Os03g26210.1	Rice Genome Annotation Project	274
HvIRO3	HORVU2Hr1G013450.14	EnsemblPlants	270
ZmIRO3	Zm00001d029339	EnsemblPlants	270
SbbHLH063	XP_021307582.1	NCBI BLASTp	271
BpbHLH063	BRADI_1g61110v3	EnsemblPlants	263
MabHLH063	XP_009386792.1	NCBI BLASTp	247
AtPYE	AT3G47640.1	TAIR Arabidopsis	240
PtbHLH047	POPTR_015G142700v3	EnsemblPlants	241
StbHLH047	PGSC0003DMG400000599	EnsemblPlants	241
SlbHLH047	Solyc03g116340.3.1	EnsemblPlants	241
MtbHLH047	MTR_4g108360	EnsemblPlants	246
PvbHLH047	XP_007155319.1	NCBI BLASTp	218
TpbHLH047	Tp57577_TGAC_v2_gene34729	EnsemblPlants	227
OsHRZ1	LOC_Os01g49470	Rice Genome Annotation Project	1236
OsHRZ2	LOC_Os05g47780	Rice Genome Annotation Project	812
HvHRZ1	HORVU3Hr1G064080.2	EnsemblPlants	1234
HvHRZ2	HORVU1Hr1G083240	IPK BLAST Server	1226
ZmHRZ1	Zm00001d043805_T001	EnsemblPlants	1233
ZmHRZ2	Zm00001d038916_T001	EnsemblPlants	1205
BdHRZ1	BRADI_2g46957v3	EnsemblPlants	1231
BdHRZ2	BRADI_2g17507v3	EnsemblPlants	1234
SbHRZ1	SORBI_3003G262200	EnsemblPlants	1272
SbHRZ2	SORBI_3009G222200	EnsemblPlants	1215
MaHRZ	GSMUA_Achr9G19220_001	Banana Genome Hub	1288
AtBTS	AT3G18290.1	TAIR Arabidopsis	1254
PtBTS	POPTR_012G055100v3	EnsemblPlants	1240
TpBTS	Tp57577_TGAC_v2_gene32705	EnsemblPlants	1236
SlBTS	Solyc05g009150.3	EnsemblPlants	1064
MtBTS	MTR_8g104410	EnsemblPlants	1243
PvBTS	PHAVU_002G317800g	EnsemblPlants	1236

Supplementary Table 3 Primers used for quantitative reverse transcription-PCR analyses.

Gene	Forward primer (5' – 3')	Reverse primer (5' – 3')	PCR product length (bp)	Annealing temperature (C°)
TaIRO3-A	ACAGGCAGACCAATGGGAAG	GCAGCTCATTCCTCTCCATC	147	64
TaIRO3-B	TTTTCCCGTCACATCAGC	GGTTCAAGGCCTTCGATGT	172	60
TaIRO3-D	AGTTTTCCCGTCACAGCAG	GCGCGTGATATGGTTGGT	211	60
TaHRZ1-A	GCAAGTCTATCCGGGTTGC	CACTCATTTAGCCACTCACCAA	259	65
TaHRZ1-B	GCACAATGTGAGCCATTCATATAC	TCGTAGCAAGCTCATTGTACTTC	203	60
TaHRZ1-D	GTTCCTTCCGTGGCTCTCAA	TGTATGCTTTATATGCCCGACTT	268	61
TaHRZ2-A	CCAGTCAAGCGAGGGAAAC	TCTGGGCTGGAAAATGTCA	254	60
TaHRZ2-B	TGGGTTACATCGGCACTTAG	CTTCCAACCAGGCTTGAACAT	216	61
TaHRZ2-D	CATATTGCTTGAGCTTTCACACTT	TCAAACAGTGGCCACAACTC	219	65
TaCyc	CAAGCCGCTGCACTACAAGG	AGGGGACGGTGCAGATGAA	227	60
TaActin	GACAATGGAACCGGAATGGTC	GTGTGATGCCAGATTTTCTCCAT	236	60
TaGAPDH	TTCAACATCATTCCAAGCAGCA	CGTAACCCAAAATGCCCTTG	220	60
TaEFA	CAGATTGGCAACGGCTACG	CGGACAGCAAAACGACCAAG	227	60
